# Characterization of CD8 + and CD68 + Microenvironment and PDL1 Expression in HPV-related Multiphenotypic Sinonasal Carcinoma

**DOI:** 10.1007/s12105-026-01908-0

**Published:** 2026-03-19

**Authors:** João Epaminondas Silva de Araújo, João Paulo Gonçalves de Paiva, Igor Lima Fernandes, Lucas Faria Abrahao-Machado, Alexandre de Oliveira Sales, Maíra Medeiros Pacheco de Andrade, Ciro Dantas Soares

**Affiliations:** 1 Oral Diagnosis Department, Piracicaba Dental School, University of Campinas (UNICAMP), Av. Limeira, 901, Piracicaba, São Paulo, Brazil; 2 Laboratório Bacchi, Private Pathology Service, Botucatu, São Paulo, 13414-903 Brazil; 3 Getúlio Sales Diagnósticos (GSD), Private Pathology Service, Natal, Rio Grande do Norte Brazil

**Keywords:** HPV-related Multiphenotypic Sinonasal Carcinoma, Tumor Microenvironment, Immune Checkpoint Inhibitors, PD L1

## Abstract

**Aim:**

To characterize the expression of CD8 + T cells, CD68 + macrophages, and PDL1 in HPV-related multiphenotypic sinonasal carcinoma (HMSC) and evaluate their correlations with clinical outcomes.

**Materials and Methods:**

This retrospective cross-sectional study analyzed 27 HMSC cases. Clinical and histopathological data were obtained from medical records. Immunohistochemical expression of CD8 and CD68 was assessed quantitatively and qualitatively in stromal and intratumoral compartments. PDL1 expression was evaluated using the Combined Positive Score (CPS). HPV genotyping was performed using the Anyplex II HPV28 assay. Statistical analyses included descriptive statistics, Fisher’s exact test, Chi-square test, Spearman’s correlation, Student’s t-test, Mann-Whitney U test, Kaplan-Meier survival analysis with log-rank test, and Cox proportional hazards models.

**Results:**

The cohort included 15 (55.6%) males and 12 (44.4%) females, with a mean age of 59.5 years. Most patients presented without recurrence (*n* = 17, 63.0%), lymph node metastasis (*n* = 22, 81.5%), or distant metastasis (*n* = 23, 85.2%). HPV-33 was the predominant genotype, detected in 17 cases (63.0%). PDL1 positivity was observed in 12 tumors (44.4%) and correlated with increased CD8 + infiltration (ρ = 0.602, *p* < 0.01). Higher densities of CD8 + T cells and CD68 + macrophages were associated with reduced recurrence risk. Older age correlated with higher Ki67 index (ρ = 0.452, *p* < 0.05), lower PDL1 expression (ρ=-0.436, *p* < 0.05), and increased recurrence. Lymph node and distant metastases were associated with poorer disease-specific survival (*p* = 0.020 and *p* = 0.010, respectively).

**Conclusions:**

The immune microenvironment, characterized by CD8 + and CD68 + cell density and PDL1 expression, together with patient age, appears to influence clinical outcomes in HMSC. These findings suggest that a subset of HMSC patients, particularly those with an inflamed tumor microenvironment, may be candidates for PDL1-targeted immunotherapy.

**Supplementary Information:**

The online version contains supplementary material available at 10.1007/s12105-026-01908-0.

## Introduction

Human papillomavirus-related multiphenotypic sinonasal carcinoma (HMSC) is a recently recognized malignant neoplasm among sinonasal tumors. HMSC occurs exclusively in the sinonasal tract, particularly the nasal cavity [1, 2]. Most cases occur in middle-aged adults, without significant sex predilection or specific symptomatology, often leading to diagnostic delays [3]. Microscopically, HMSC exhibits substantial overlap with salivary gland-type and neoplasms of the sinonasal surface epithelium, representing a considerable diagnostic challenge for pathologists [4].

The genotypic profile of HMSC is predominantly characterized by high-risk HPV subtypes, with HPV-33 being the most frequently detected, followed by HPV-35, HPV-16, and HPV-52 [2, 5]. HMSC presents unique therapeutic challenges owing to its rarity and distinct relapsing clinical behavior [1].

The tumor microenvironment plays a pivotal role in cancer progression, therapeutic response, and overall outcomes. In head and neck carcinomas, it represents a highly complex system involving dynamic interactions between immune cells, stromal components, and malignant cells. A central element is the presence of tumor-infiltrating lymphocytes (TILs), particularly CD8 + T cells, whose functional status and spatial distribution have a significant impact on treatment efficacy and patient prognosis [6, 7]. One of the most relevant mechanisms within this microenvironment is the Programmed Death-Ligand (PDL1) pathway, which enables tumors to evade immune surveillance. In sinonasal carcinomas, PDL1 expression is markedly heterogeneous across different histological subtypes and often correlates with TIL density, supporting its potential utility as a predictive biomarker for immunotherapy [8, 9].

The efficacy of immune checkpoint inhibitors, such as pembrolizumab and nivolumab, is well-established in common head and neck carcinomas, with response rates frequently correlating with PDL1 expression [10, 11]. Emerging evidence indicates that this therapeutic strategy may also be beneficial in rare sinonasal carcinomas, with case reports and clinical studies demonstrating promising outcomes for pembrolizumab in combination with chemotherapy across distinct tumors [12, 13]. This observation is particularly relevant since HPV-positive tumors, including a subset of sinonasal carcinomas, often display distinct immune profiles and enhanced responsiveness to immunotherapy when compared with HPV-negative [14, 15].

However, substantial knowledge gaps persist regarding the rare entity of HMSC. Its low incidence has limited comprehensive investigations, leaving the associations between HPV subtypes, PDL1 expression patterns, and TIL composition largely undefined. A systematic evaluation of these immune microenvironmental features is essential for advancing personalized therapeutic strategies, refining patient selection for immunotherapy, improving prognostic stratification, and elucidating the unique biological behavior of this tumor.

Therefore, the present study aims to characterize the inflammatory microenvironment and PDL1 expression patterns in HMSC, as well as to assess their correlations with prognostic outcomes. By analyzing tumor-infiltrating lymphocyte populations and immune checkpoint expression, this investigation seeks to expand the current understanding of HMSC immune biology and provide a foundation for future therapeutic approaches in this rare but clinically significant malignancy.

## Materials and Methods

This retrospective cross-sectional study was conducted following the principles outlined in the Declaration of Helsinki and was approved by the Research Ethics Committee under protocol CAAE 76629323.4.0000.5418 (CEP/CONEP Brazil). All procedures involving human participants were performed in accordance with institutional ethical standards and national research regulations.

### Case Selection

Cases of HMSC were identified from the archives of Getulio Sales Diagnósticos and Hospital Herrera Llerandi in Guatemala, covering a 23-year period from January 2001 to December 2024. An initial cohort was systematically reviewed by two experienced head and neck pathologists to confirm the HMSC diagnosis based on established morphological and immunohistochemical parameters [1, 2]. Inclusion criteria required a histologically confirmed HMSC diagnosis, adequate formalin-fixed paraffin-embedded (FFPE) tissue blocks for immunohistochemical and molecular analysis, sufficient clinical and follow-up data, and positive p16 immunohistochemical staining (defined as > 70% nuclear and cytoplasmic positivity) [1]. Exclusion criteria consisted of cases with insufficient tissue for a comprehensive immunohistochemical panel, inadequate clinical documentation or missing follow-up information, cases with equivocal histological features that precluded a definitive HMSC diagnosis, and any previously treated cases where neoadjuvant therapy might have altered the immune microenvironment. After applying the inclusion and exclusion criteria, 27 cases were selected for final analysis.

Comprehensive clinical and histopathological information was systematically extracted from medical records and pathology reports. The collected data encompassed patient demographics (age at diagnosis, sex), tumor characteristics (anatomical location, size in centimeters), staging (according to the American Joint Committee on Cancer 8th edition TNM system), treatment modalities, clinical outcomes (recurrence, lymph node metastasis, distant metastasis), and follow-up status. All hematoxylin and eosin (H&E) stained sections were reviewed independently by two experienced head and neck pathologists to confirm the diagnosis and assess morphological features. Discrepant cases were resolved through consensus review. The histological evaluation included quantification of epithelial dysplasia thickness (in layers), mitotic count per 2 mm², and the assessment of the presence or absence of pleomorphism, necrosis, perineural invasion (PNI), and vascular invasion (VI). Furthermore, the tumor’s cytological appearance (basophilic, clear, spindle, eosinophilic), nuclear features, architectural growth patterns (solid, tubular, cords, cribriform, etc.), and stromal characteristics (myxoid, hyaline, myxohyaline) were documented.

## Immunohistochemistry

Immunohistochemical staining was performed on 4-µm thick sections using the automated Agilent/Dako Omnis platform. The following pre-diluted primary antibodies (Agilent/Dako) were employed: CD8 (cytotoxic T-cell marker), CD68 (macrophage cell marker), and PDL1 (clone PD-L1 IHC 22C3 pharmDx). Antigen retrieval was performed using the appropriate retrieval solutions according to the manufacturer’s protocols optimized for each antibody. PDL1 expression was evaluated using the Combined Positive Score (CPS), which encompasses both tumor cells and tumor-associated immune cells.

The CPS was calculated as the number of PDL1 staining cells (tumor cells, lymphocytes, macrophages) divided by the total number of viable tumor cells, multiplied by 100. A cut-off value of > 5% was established for positivity, with squamous cell carcinoma of the lung serving as positive control tissue. CD8 + and CD68 + cells were quantified in both intraepithelial and stromal compartments, with cell density assessed using a standardized scoring system and separate evaluation of peritumoral and intratumoral distributions.

Although CPS values are conventionally reported as integer categories (< 1, ≥ 1, etc.) in clinical practice and literature, our digital quantification method enabled precise measurement of PDL1-positive tumor cells, PDL1-positive inflammatory cells, and total viable cells within each region of interest. Consequently, we report CPS as continuous values derived from these exact counts. For analytical purposes, a binary cutoff of CPS ≥ 5 was applied to define PDL1 positivity, consistent with established thresholds in other carcinomas. All cases classified as positive in our cohort had CPS values ≥ 5, with some exceeding 30. This approach aligns with recent methodological observations that, while CPS is not typically reported as a fraction, underlying quantitative assessment may yield continuous scores that reflect the actual proportion of PDL1-expressing cells [16].

## Digital Image Analysis

Digital image analysis was performed using ImageScope software (Leica Biosystems). For each case, 10 representative high-power fields (×400 magnification) were systematically selected by two experienced pathologists, focusing on areas with the highest immune cell infiltration and avoiding necrotic or poorly preserved regions. Regions of interest (ROI) were carefully delineated to ensure standardized measurement across all cases. Quantitative analysis included total lymphocyte counts per high-power field, CD8+/CD68 + ratio calculation, PDL1 positive cell density, and spatial distribution assessment of immune markers.

## HPV Genotyping

Genomic DNA was extracted from FFPE tissue sections using standard protocols optimized for archived specimens, with DNA quality and quantity assessed before molecular analysis to ensure an adequate template for PCR amplification. HPV detection and genotyping were performed using the Anyplex™ II HPV28 Detection kit (Seegene), which enables simultaneous detection and genotyping of 28 HPV types including 19 high-risk types (16, 18, 26, 31, 33, 35, 39, 45, 51, 52, 53, 56, 58, 59, 66, 68a, 68b, 73, 82) and 9 low-risk types (6, 11, 40, 42, 43, 44, 54, 61, 70). The assay utilizes real-time PCR with melting curve analysis for accurate genotype identification. Quality controls included HPV-positive oropharyngeal squamous cell carcinoma with confirmed p16 positivity as a positive control, p16-negative basal cell carcinoma of skin as a negative control, and β-globin gene amplification as an internal control to verify DNA integrity.

### Statistical Analysis

Statistical analyses were performed using R Studio (Version 4.3.0). Descriptive statistics were calculated for all variables, with continuous variables presented as means ± standard deviations or medians with interquartile ranges, depending on data distribution as assessed by Shapiro-Wilk normality tests. Categorical variables were presented as frequencies and percentages. Associations between categorical variables were evaluated using Fisher’s exact test or chi-square test, as appropriate, based on expected cell counts. Correlations between continuous and ordinal variables were assessed using Spearman’s rank correlation coefficient. Comparisons of continuous variables between two independent groups were performed using Student’s t-test for normally distributed data or the Mann-Whitney U test for non-normally distributed data. Survival analyses were performed using Kaplan-Meier methodology with Log-Rank tests for group comparisons. Hazard ratios and 95% confidence intervals were calculated using Cox proportional hazards regression models. The proportional hazards assumption for Cox models was verified using Schoenfeld residuals. Multivariable models were constructed by including variables with *p* < 0.10 in univariate analysis or those deemed clinically relevant, but were limited to two predictors at a time to avoid overfitting. All statistical tests were two-sided, and *p*-values < 0.05 were considered statistically significant.

## Results

The complete descriptive analysis of demographic, clinicopathological, treatment, prognostic, and immunohistochemical features of 27 HMSC patients included in this study is presented in Supplementary Tables 1, 2 and 3, and Table 1 (Table 1).

**Table 1:** Clinical, histopathological, HPV genotyping, inflammatory microenvironment, and prognostic features of HMSC patients included in this study

Characteristic	Value	Characteristic	Value
Demographics		Histopathology	
Age (years), mean ± SD	59.5 ± 18.3	Pleomorphism, n (%)	
Sex, n (%)		Present	25 (92.6)
Female	15 (55.6)	Absent	2 (7.4)
Male	12 (44.4)	Necrosis, n (%)	
Tumor features		Present	20 (74.1)
Location, n (%)		Absent	7 (25.9)
Paranasal sinus (NOS)	14 (51.9)	Vascular invasion, n (%)	
Nasal cavity (NOS)	13 (48.1)	Present	3 (11.1)
TNM stage, n (%): T3N0M0/T2N0M0/T1N0M0/T4N0M0	14 (51.9)/ 6 (22.2)/ 4 (14.8)/ 3 (11.1)	Absent	24 (88.9)
Tumor size (cm), mean ± SD	4.24 ± 2.13	Cell appearance, n (%):	
**Treatment**,** n (%)**		Basophilic	24 (88.9)
Surgery + Radiotherapy	15 (55.6)	Clear	22 (81.5)
Surgery alone	8 (29.6)	Spindle	21 (77.8)
Surgery + Chemoradiotherapy	3 (11.1)	Eosinophilic	11 (40.7)
Radiotherapy alone	1 (3.7)	Nuclear appearance, n (%):	
**HPV status**		Dispersed chromatin	11 (40.7)
HPV infection pattern, n (%)		Hyperchromatic, indistinct nucleoli	8 (29.6)
Single HPV type	22 (81.5)	Hyperchromatic, small, distinct nucleoli	8 (29.6)
HPV coinfection	5 (18.5)	Architecture‡, n (%):	
Single HPV type (*n* = 22):		Solid	11 (40.7)
HPV 33	17 (63.0)	Tubular	9 (33.3)
HPV 35	3 (11.1)	Cords	9 (33.3)
HPV 16	1 (3.7)	Cribriform	9 (33.3)
HPV 52	1 (3.7)	Single cells	4 (14.8)
HPV coinfection (*n* = 5):		Ribbon-like	2 (7.4)
HPV 33 + 51	2 (7.4)	Nests	1 (3.7)
HPV 16 + 33	1 (3.7)	Glomeruloid-like	1 (3.7)
HPV 26 + 33	1 (3.7)	Stroma, n (%):	
HPV 18 + 52	1 (3.7)	Myxoid	11 (40.7)
**Clinical outcomes**		Hyaline	10 (37.0)
Recurrence, n (%)		Myxohyaline	6 (22.2)
Absent	17 (63.0)	Immunohistochemistry	
Present	10 (37.0)	PDL1 expression (CPS ≥ 5), n (%)	
Time to recurrence (months), median (range)†	15.5 (6–31)	Negative	15 (55.6)
Lymph node metastasis (LNM), n (%)		Positive	12 (44.4)
Absent	22 (81.5)	CPS, median (IQR)	0.7 (0.0-19.1)
Present	5 (18.5)	CPS distribution, n (%)	
Time to LNM (months), median (range)†	14 (3–29)	CPS 0	9 (33.3)
Distant metastasis (DM), n (%)		CPS 0.1–4.9	6 (22.2)
Absent	23 (85.2)	CPS 5–19	6 (22.2)
Present	4 (14.8)	CPS ≥ 20	6 (22.2)
Time to DM (months), median (range)†	19 (3–48)	CD8 count, median (IQR)	453 (403–789)
Sites of distant metastasis‡:		CD8 median, n (%)	
Lung	2 (50.0)	High (≥ median)	14 (51.9)
Liver	1 (25.0)	Low (< median)	13 (48.1)
Bone	1 (25.0)	CD68 count, mean ± SD	370.1 ± 137.6
**Follow-up**		CD68 median, n (%)	
Follow-up status, n (%)		High (≥ median)	14 (51.9)
Alive without disease	23 (85.2)	Low (< median)	13 (48.1)
Alive with disease	2 (7.4)	Ki67 fraction, median (IQR)	0.57 (0.32–0.68)
Dead of disease	2 (7.4)	Ki67 split, n (%)	
Overall follow-up (months), median (range)	43 (5-101)	High (≥ median)	14 (51.9)
**Histopathology (cont.)**		Low (< median)	13 (48.1)
Epithelial dysplasia (layers), median (range)	8 (3–20)		
Mitoses (/2 mm²), median (IQR)	7 (5–11)		
Perineural invasion, n (%)			
Present	4 (14.8)		
Absent	23 (85.2)		

SD: standard deviation; IQR: interquartile range; NOS: not otherwise specified

*Percentages for cellular features (cell appearance, architecture) sum to > 100% as cases often show multiple patterns simultaneously.

†Time intervals calculated only among cases with the event (recurrence: *n* = 10; LNM: *n* = 5; DM: *n* = 4).

‡Sites of distant metastasis percentages calculated from the 4 cases with distant metastasis. Continuous variables presented as mean ± SD for normally distributed variables or median (IQR/range) for non-normally distributed variables based on Shapiro-Wilk test. All percentages calculated from total cohort (*n* = 27) unless otherwise specified

The mean age at presentation was 59.5 ± 18.3 years, with a nearly equal sex distribution (15 females, 55.6%; 12 males, 44.4%). Tumors were located in the paranasal sinuses (14 cases, 51.9%) and nasal cavity (13 cases, 48.1%), with a mean size of 4.24 ± 2.13 cm. Most patients presented with advanced local disease (T3/T4 stage: 17 cases, 63.0%).

Treatment approaches included surgery with adjuvant radiotherapy for the majority of patients (15 cases, 55.6%), followed by surgery alone (8 cases, 29.6%), surgery with chemoradiotherapy (3 cases, 11.1%), and radiotherapy alone (1 case, 3.7%).

HPV genotyping revealed that most tumors harbored a single HPV type (22 cases, 81.5%). Among these, HPV-33 was the predominant genotype, identified in 17 cases (63.0% of the entire cohort). Other single infections included HPV-35 (3 cases, 11.1%), HPV-16 (1 case, 3.7%), and HPV-52 (1 case, 3.7%). Coinfections with two HPV types were present in 5 cases (18.5%), with the most frequent combination being HPV-33 and HPV-51 (2 cases, 7.4%). The remaining coinfections were HPV-16 + HPV-33, HPV-26 + HPV-33, and HPV-18 + HPV-52, each found in one case (3.7%).

Clinical outcomes revealed that local recurrence developed in 10 patients (37.0%), with a median time to recurrence of 15.5 months (range: 6–31 months). Lymph node metastasis was less common, occurring in 5 patients (18.5%) after a median of 14 months (range: 3–29 months). Distant metastasis was the least frequent event, observed in 4 patients (14.8%) with a median onset of 19 months (range: 3–48 months). Among these four cases, the lung was the most common site of distant spread (2 cases, 50.0%), followed by the liver and bone (1 case each, 25.0%). At the last follow-up, most patients were alive (25 cases, 92.6%), with 23 (85.2%) being alive without evidence of disease and 2 (7.4%) alive with active disease. Two patients (7.4%) had died from HMSC. The median overall follow-up time for the cohort was 43 months, ranging from 5 to 101 months.

Histopathological evaluation revealed variable dysplasia of the overlying sinonasal epithelium, ranging from 3 to 20 cell layers in thickness (median: 8 layers). Tumor proliferative activity was moderate, with a median mitotic count of 7 per 2 mm² (IQR: 5–11). The neoplastic cells showed marked pleomorphism in most cases (25 tumors, 92.6%), and necrosis was frequently present (20 tumors, 74.1%). In contrast, perineural invasion was identified in 4 cases (14.8%) and vascular invasion in 3 cases (11.1%). The tumors were histologically complex, typically demonstrating multiple cellular morphologies, nuclear patterns, and architectural arrangements within the same lesion. At the cellular level, basaloid cells were the most prevalent feature, identified in 24 cases (88.9%), followed by clear cells (22 cases, 81.5%) and spindle cells (21 cases, 77.8%). Eosinophilic cells were present in a smaller subset of 11 tumors (40.7%). Nuclear features included dispersed chromatin (11 cases, 40.7%), hyperchromatic nuclei with indistinct nucleoli (8 cases, 29.6%), and hyperchromatic nuclei with small but distinct nucleoli (8 cases, 29.6%). Tumor stroma was myxoid (11 cases, 40.7%), hyaline (10 cases, 37%), or myxohyaline (6 cases, 22.2%).

**Fig. 1:**
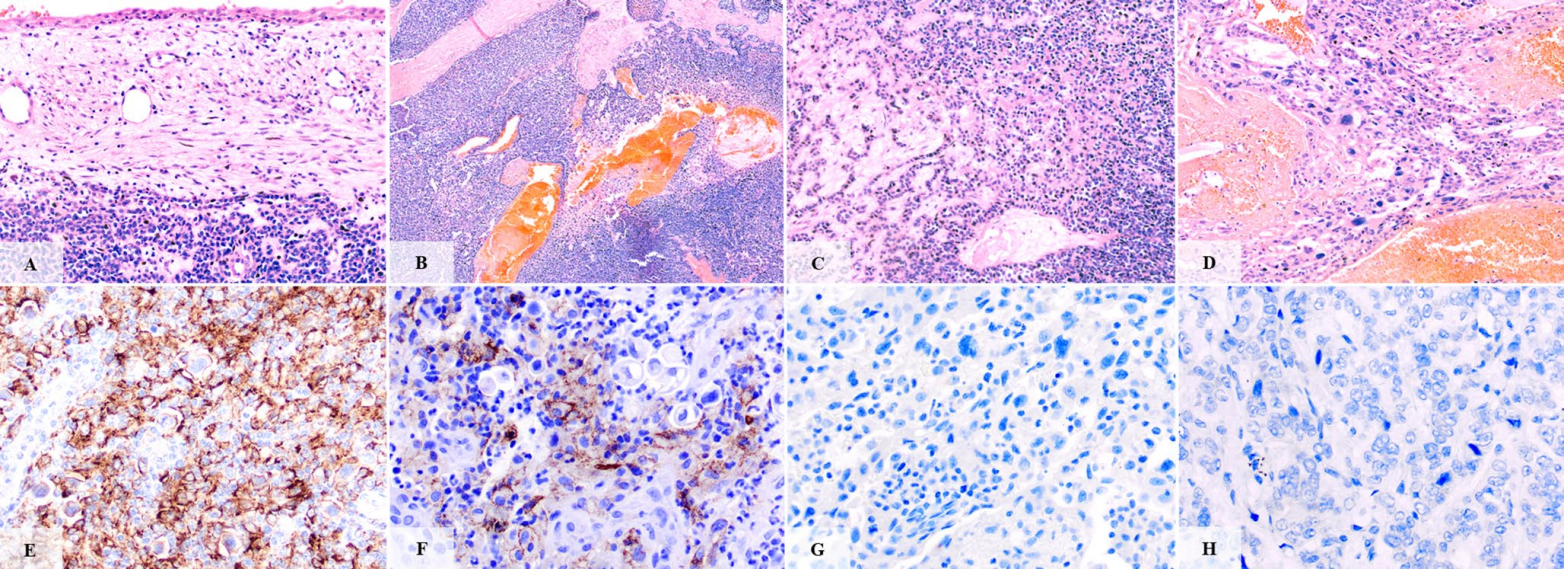
Representative histopathological features and PDL1 expression in HMSC. A 2-layered sinonasal epithelium with dysplasia is shown. Note the proliferation of highly basaloid cells adjacent to the epithelium (**A**). Most of the tumor was arranged in solid proliferation and presented hemorrhagic and necrotic areas (**B**). Areas with nested/cord arrangements within a highly hyaline stroma (**C**). Tumor cells with bizarre anaplasia (**D**). Immunohistochemical staining for PDL1 showing (**E**, **F**) PDL1-positive tumors (CPS ≥ 5) and (**G**, **H**) PDL1-negative tumors (CPS < 5)

**Fig. 2:**
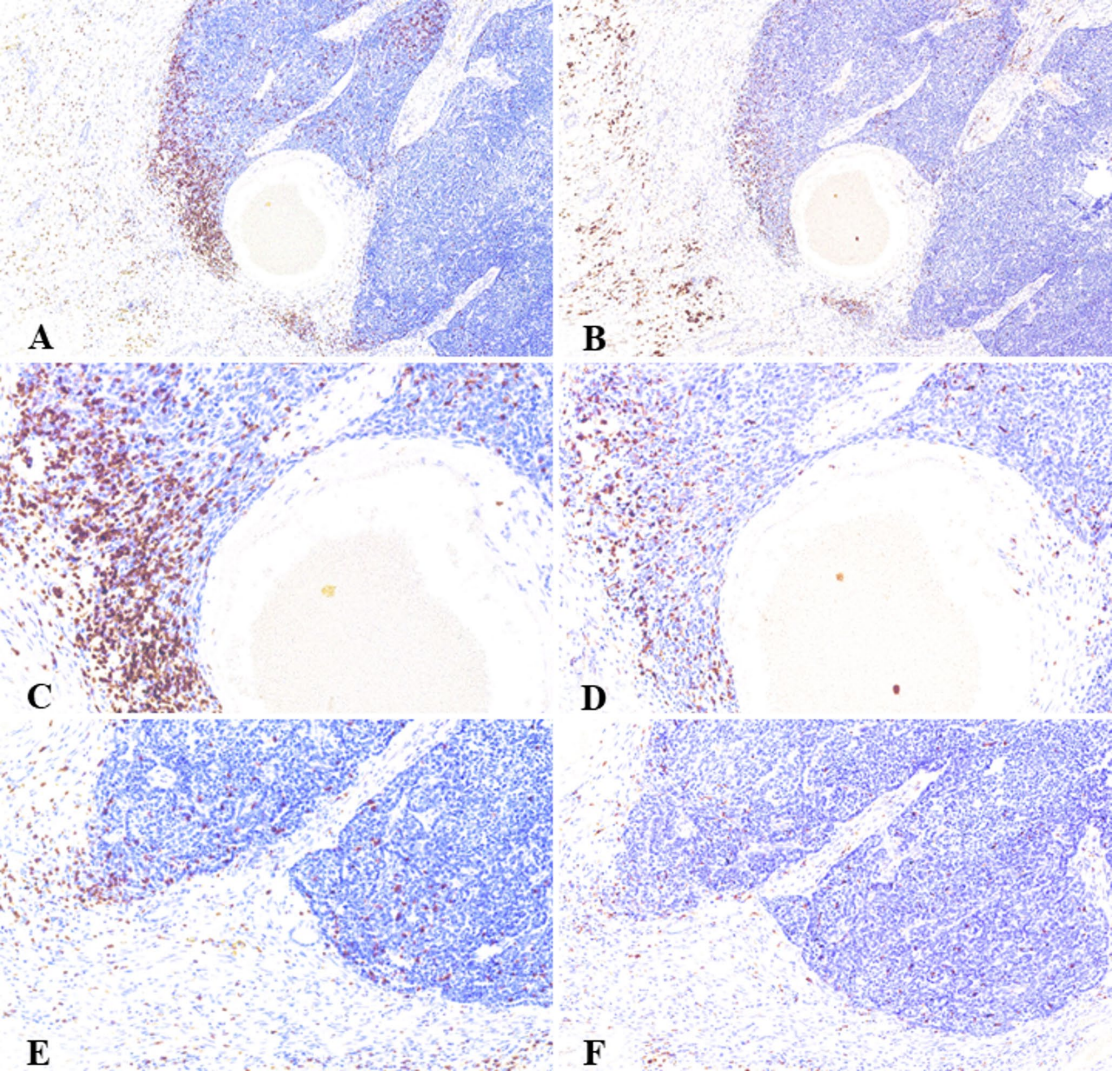
Density and distribution of CD8 + and CD68 + cells in HMSC. **A**, **C**, **E** CD8 + T-cells localize to both the stromal and intratumoral compartments. **B**, **D**, **F** CD68 + cells show a similar distribution pattern

Immunohistochemical characterization of the tumor microenvironment showed that PDL1 expression, using a combined positive score (CPS) cutoff of ≥ 5, was positive in 12 tumors (44.4%) and negative in 15 (55.6%). The median CPS across the cohort was 0.7 (IQR: 0.0–19.1), with a broad distribution: 9 tumors (33.3%) had a CPS of 0, 6 (22.2%) scored between 0.1 and 4.9, another 6 (22.2%) between 5 and 19, and 6 (22.2%) had a CPS of 20 or higher (Fig. 1). The median density of CD8 + cytotoxic T lymphocytes was 453 cells (IQR: 403–789), while the mean density of CD68 + macrophages was 370.1 ± 137.6 cells (Fig. 2). The median Ki67 proliferative index was 0.57 (IQR: 0.32–0.68). For analytical purposes, each of these three markers was dichotomized at its respective median value. This resulted in nearly equal cohort splits, with 14 patients (51.9%) classified as “high” and 13 patients (48.1%) as “low” for CD8 + density, CD68 + density, and Ki67 index.

Statistical analyses using Chi-square or Fisher’s exact tests identified significant associations between clinicopathological variables and clinical outcomes. Male sex showed a significant association with the absence of lymph node metastasis (*p =* 0.047). Regarding local recurrence, positive PDL1 expression was more frequently observed in recurrence-free patients (*p =* 0.014). Similarly, the high density of CD8 + T-cells (above median) was more common in patients without recurrence (*p* = 0.018), as was a high density of CD68 + macrophages (*p =* 0.018). Patients who were alive without disease were significantly more likely to be free of lymph node metastasis (*p =* 0.020) and distant metastasis (*p =* 0.010) than those who died from the disease. Finally, the development of distant metastasis was strongly linked to lymph node status. Patients without distant metastasis had a significantly higher frequency of being free from lymph node involvement compared to those with distant metastasis (*p* < 0.001) (Table 2).

**Table 2:** Association tests between clinicopathological variables and prognostic outcomes

Variable	DSS Alive *N* (%)	DSS Dead *N* (%)	*p*-value	Recurrence No *N* (%)	Recurrence Yes *N* (%)	*p*-value	LN Metastasis No *N* (%)	LN Metastasis Yes *N* (%)	*p*-value	Distant Metastasis No *N* (%)	Distant Metastasis Yes *N* (%)	*p*-value
Sex												
Female	12 (48.0)	2 (8.0)	0.487	9 (33.3)	6 (22.2)	1.000	10 (37.0)	5 (18.5)	0.047	11 (40.7)	4 (14.8)	0.106
Male	11 (44.0)	0 (0.0)		8 (29.6)	4 (14.8)		12 (44.4)	0 (0.0)		12 (44.4)	0 (0.0)	
**Location**												
Paranasal sinus	12 (48.0)	1 (4.0)	1.000	7 (25.9)	7 (25.9)	0.236	11 (40.7)	3 (11.1)	1.000	12 (44.4)	2 (7.4)	1.000
Nasal cavity	11 (44.0)	1 (4.0)		10 (37.0)	3 (11.1)		11 (40.7)	2 (7.4)		11 (40.7)	2 (7.4)	
**HPV genotyping**												
Single HPV-type	19 (76.0)	1 (4.0)	0.367	12 (44.4)	10 (37.0)	0.124	19 (70.4)	3 (11.1)	0.221	20 (74.1)	2 (7.4)	0.144
HPV coinfection	4 (16.0)	1 (4.0)		5 (18.5)	0 (0.0)		3 (11.1)	2 (7.4)		3 (11.1)	2 (7.4)	
**Ki67 split**												
< Median	12 (48.0)	0 (0.0)	0.480	10 (37.0)	3 (11.1)	0.236	11 (40.7)	2 (7.4)	1.000	12 (44.4)	1 (3.7)	0.569
> Median	11 (44.0)	2 (8.0)		7 (25.9)	7 (25.9)		11 (40.7)	3 (11.1)		11 (40.7)	3 (11.1)	
**PDL1 expression**												
Negative	12 (48.0)	1 (4.0)	1.000	6 (22.2)	9 (33.3)	0.014	13 (48.1)	2 (7.4)	0.628	13 (48.1)	2 (7.4)	1.000
Positive	11 (44.0)	1 (4.0)		11 (40.7)	1 (3.7)		9 (33.3)	3 (11.1)		10 (37.0)	2 (7.4)	
**CD8 median**												
< Median	11 (44.0)	1 (4.0)	1.000	5 (18.5)	8 (29.6)	0.018	11 (40.7)	2 (7.4)	1.000	11 (40.7)	2 (7.4)	1.000
> Median	12 (48.0)	1 (4.0)		12 (44.4)	2 (7.4)		11 (40.7)	3 (11.1)		12 (44.4)	2 (7.4)	
**CD68 median**												
< Median	10 (40.0)	1 (4.0)	1.000	5 (18.5)	8 (29.6)	0.018	11 (40.7)	2 (7.4)	1.000	11 (40.7)	2 (7.4)	1.000
> Median	13 (52.0)	1 (4.0)		12 (44.4)	2 (7.4)		11 (40.7)	3 (11.1)		12 (44.4)	2 (7.4)	
**TNM Grouped**												
T1/T2	9 (36.0)	1 (4.0)	1.000	7 (25.9)	3 (11.1)	0.692	8 (29.6)	2 (7.4)	1.000	9 (33.3)	1 (3.7)	1.000
T3/T4	14 (56.0)	1 (4.0)		10 (37.0)	7 (25.9)		14 (51.9)	3 (11.1)		14 (51.9)	3 (11.1)	
**Pleomorphism**												
Yes	21 (84.0)	2 (8.0)	1.000	16 (59.3)	9 (33.3)	1.000	20 (74.1)	5 (18.5)	1.000	21 (77.8)	4 (14.8)	1.000
No	2 (8.0)	0 (0.0)		1 (3.7)	1 (3.7)		2 (7.4)	0 (0.0)		2 (7.4)	0 (0.0)	
**Necrosis**												
Yes	18 (72.0)	1 (4.0)	0.430	12 (44.4)	8 (29.6)	0.678	17 (63.0)	3 (11.1)	0.580	18 (66.7)	2 (7.4)	0.269
No	5 (20.0)	1 (4.0)		5 (18.5)	2 (7.4)		5 (18.5)	2 (7.4)		5 (18.5)	2 (7.4)	
**PNI**												
Yes	4 (16.0)	0 (0.0)	1.000	2 (7.4)	2 (7.4)	0.613	3 (11.1)	1 (3.7)	1.000	4 (14.8)	0 (0.0)	1.000
No	19 (76.0)	2 (8.0)		15 (55.6)	8 (29.6)		19 (70.4)	4 (14.8)		19 (70.4)	4 (14.8)	
**VI**												
Yes	3 (12.0)	0 (0.0)	1.000	2 (7.4)	1 (3.7)	1.000	2 (7.4)	1 (3.7)	0.474	3 (11.1)	0 (0.0)	1.000
No	20 (80.0)	2 (8.0)		15 (55.6)	9 (33.3)		20 (74.1)	4 (14.8)		20 (74.1)	4 (14.8)	
**Treatment group**												
Surgery+adjuvant	16 (64.0)	2 (8.0)	1.000	13 (48.1)	6 (22.2)	0.415	14 (51.9)	5 (18.5)	0.280	15 (55.6)	4 (14.8)	0.285
Surgery alone	7 (28.0)	0 (0.0)		4 (14.8)	4 (14.8)		8 (29.6)	0 (0.0)		8 (29.6)	0 (0.0)	
**Recurrence**												
No	16 (64.0)	1 (4.0)	1.000	–	–	–	14 (51.9)	3 (11.1)	1.000	15 (55.6)	2 (7.4)	0.613
Yes	7 (28.0)	1 (4.0)		–	–	–	8 (29.6)	2 (7.4)		8 (29.6)	2 (7.4)	
**LN Metastasis**												
No	21 (84.0)	0 (0.0)	0.020	14 (51.9)	3 (11.1)	1.000	–	–	–	22 (81.5)	0 (0.0)	< 0.001
Yes	2 (8.0)	2 (8.0)		8 (29.6)	2 (7.4)		–	–	–	1 (3.7)	4 (14.8)	
**Distant Metastasis**												
No	22 (88.0)	0 (0.0)	0.010	15 (55.6)	8 (29.6)	0.613	22 (81.5)	1 (3.7)	< 0.001	—	—	—
Yes	1 (4.0)	2 (8.0)		2 (7.4)	2 (7.4)		0 (0.0)	4 (14.8)		—	—	—

DSS, disease-specific survival; LN, lymph node; HPV, human papillomavirus; PNI, perineural invasion; VI, vascular invasion; CPS, Combined Positive Score

*p*-values were calculated using Fisher’s exact test or Chi-square test, as appropriate. Percentages are based on the total number of patients in each outcome group (*n* = 27 for the cohort; denominators for individual outcomes may vary as indicated in the text). “DSS Alive” includes patients alive without disease and alive with disease (total *n* = 25); “DSS Dead” indicates death from HMSC (*n* = 2)

Spearman correlation analysis was performed to assess the strength and direction of monotonic relationships between clinical, immunological, and histopathological variables with clinical outcomes. For local recurrence, several significant correlations were identified. Older patient age demonstrated a positive correlation with recurrence (ρ = 0.571, *p* = 0.002). Similarly, a higher Ki67 proliferative index showed a positive correlation with recurrence (ρ = 0.409, *p* = 0.034). In contrast, higher densities of CD8 + T-cells (ρ = -0.443, *p* = 0.021) and CD68 + macrophages (ρ = -0.561, *p* = 0.002), as well as positive PDL1 expression (ρ = -0.532, *p* = 0.004) and a higher CPS (ρ = -0.542, *p* = 0.003) were negatively correlated with recurrence. Correlations with DSS should be interpreted with extreme caution due to the low number of events (*n* = 2) (Table 3).

**Table 3:** Spearman rank correlation analysis of associations between clinicopathological variables and clinical outcomes

Category & Variable	Recurrence	LN Metastasis	Distant Metastasis	DSS†
	ρ (*p*-value)	ρ (*p*-value)	ρ (*p*-value)	ρ (*p*-value)
**Clinical**				
Age (years)	0.571 (0.002)	0.190 (0.343)	0.033 (0.868)	0.256 (0.217)
Tumor size (cm)	0.207 (0.300)	0.166 (0.409)	0.335 (0.087)	0.082 (0.697)
TNM Stage				
T3/T4	0.112 (0.579)	-0.029 (0.885)	0.104 (0.606)	-0.060 (0.775)
T1/T2	-0.112 (0.579)	0.029 (0.885)	-0.104 (0.606)	0.060 (0.775)
Treatment				
Surgery + Adjuvant	-0.158 (0.440)	0.325 (0.105)	0.284 (0.159)	0.193 (0.365)
Surgery only	0.158 (0.440)	-0.325 (0.105)	-0.284 (0.159)	-0.193 (0.365)
HPV status				
Coinfection (vs. single HPV)	-0.366 (0.061)	0.264 (0.184)	0.338 (0.085)	0.221 (0.288)
**Immunological**				
Ki67 fraction (%)	0.409 (0.034)	-0.043 (0.832)	0.114 (0.572)	0.266 (0.199)
CD8 + T-cell count	-0.443 (0.021)	0.141 (0.484)	0.033 (0.868)	-0.102 (0.627)
CD68 + count	-0.561 (0.002)	-0.037 (0.856)	-0.040 (0.842)	0.082 (0.698)
Mitoses (/2 mm²)	0.030 (0.883)	0.043 (0.830)	-0.088 (0.663)	0.248 (0.232)
PD-L1 status				
Positive	-0.532 (0.004)	0.149 (0.458)	0.047 (0.817)	0.012 (0.955)
Negative	0.532 (0.004)	-0.149 (0.458)	-0.047 (0.817)	-0.012 (0.955)
CPS (Combined Positive Score)	-0.542 (0.003)	0.175 (0.383)	0.014 (0.946)	-0.021 (0.922)
**Histopathological**				
Pleomorphism				
Present	0.076 (0.707)	-0.135 (0.502)	-0.118 (0.558)	-0.087 (0.679)
Absent	-0.076 (0.707)	0.135 (0.502)	0.118 (0.558)	0.087 (0.679)
Necrosis				
Present	-0.104 (0.607)	0.153 (0.446)	0.229 (0.250)	0.180 (0.391)
Absent	0.104 (0.607)	-0.153 (0.446)	-0.229 (0.250)	-0.180 (0.391)
Perineural Invasion				
Present	-0.112 (0.578)	-0.070 (0.730)	0.174 (0.386)	0.129 (0.540)
Absent	0.112 (0.578)	0.070 (0.730)	-0.174 (0.386)	-0.129 (0.540)
Vascular Invasion				
Present	0.027 (0.893)	-0.135 (0.502)	0.147 (0.463)	0.109 (0.604)
Absent	-0.027 (0.893)	0.135 (0.502)	-0.147 (0.463)	-0.109 (0.604)

LN, lymph node; DSS, disease-specific survival; CPS, Combined Positive Score.

*n* = 27 for most correlations except: DSS (*n* = 25), Treatment (*n* = 26).

† DSS: Disease-specific survival. Interpret with caution: Only 2 events (deaths from disease) were observed.

*p*-values for DSS correlations should be interpreted with extreme caution due to the low number of events.

Correlations for DSS should be interpreted with extreme caution due to the limited number of events (*n* = 2 deaths from disease). Sample sizes vary: *n* = 27 for correlations with Recurrence, LN Metastasis, and Distant Metastasis; *n* = 25 for DSS correlations (excluding two patients who died from other causes); *n* = 26 for correlations involving Treatment (one patient received radiotherapy alone).

To quantify the differences suggested by the correlation analysis, univariate comparisons of continuous variables were performed between patient groups stratified by clinical outcome. Patients who developed recurrence tended to be older (72.2 ± 9.2 years) than those who did not (52.0 ± 18.2 years; *p* = 0.001). They also showed lower mean CD68 + macrophage density (274.7 ± 120.4 vs. 426.2 ± 116.5; *p* = 0.005) and lower median CD8 + T-cell density (425 vs. 743; *p* = 0.024). The median Ki67 proliferative index was also higher in the recurrence group (0.62 vs. 0.36; *p* = 0.035). In contrast, no significant differences were found for these variables when comparing groups based on lymph node metastasis, distant metastasis, or disease-specific survival (all *p* > 0.05) (Table 4).

**Table 4:** Univariate comparisons of continuous clinicopathological and immunological variables stratified by clinical outcome

Variable	No Recurrence (*n* = 17)	Recurrence (*n* = 10)	*p*-value	No LN Metastasis (*n* = 22)	LN Metastasis (*n* = 5)	*p*-value	No Distant Metastasis (*n* = 23)	Distant Metastasis (*n* = 4)	*p*-value	Alive (*n* = 23)	Dead (*n* = 2)	*p*-value
Age (years)	52.0 ± 18.2	72.2 ± 9.2	0.001	58.0 ± 16.7	66.0 ± 25.2	0.387	59.39 ± 17.64	60.00 ± 24.62	0.952	59.39 ± 17.64	60.00 ± 24.62	0.210
Tumor size (cm)	4.0 ± 2.1	4.7 ± 2.2	0.448	4.03 ± 1.78	5.13 ± 3.40	0.304	3.91 ± 1.83	6.09 ± 3.04	0.057	3.91 ± 1.83	6.09 ± 3.04	0.688
CD68 count	426.2 ± 116.5	274.7 ± 120.4	0.005	371.6 ± 137.0	363.2 ± 156.2	0.904	372.30 ± 133.89	357.25 ± 179.67	0.844	372.30 ± 133.89	357.25 ± 179.67	0.689
CD8 count	743 (410–870)	425 (338–454)	0.024	452 (397.5-745.3)	855 (318–905)	0.473	453 (403–752)	632 (272.5-923.3)	0.864	453 (406-770.5)	541 (227–855)	0.616
Ki67 fraction	0.36 (0.22–0.65)	0.62 (0.44–0.70)	0.035	0.51 (0.34–0.67)	0.67 (0.09–0.69)	0.851	0.46 (0.32–0.67)	0.68 (0.21–0.69)	0.539	0.46 (0.31–0.65)	0.68 (0.67–0.68)	0.193
Mitoses/2 mm²	7 (5–11)	7 (5–11)	0.879	7 (5–11)	11 (4-12.5)	0.825	7 (5–11)	8 (3.5–11)	0.654	7 (5.5–11)	11 (11–11)	0.224

Data are presented as mean ± standard deviation for normally distributed variables (Age, Tumor size, CD68 count) and as median (interquartile range) for non-normally distributed variables (CD8 count, Ki67 fraction, Mitoses).

*p*-values were calculated using Student’s t-test for normally distributed variables and the Mann-Whitney U test for non-normally distributed variables. Abbreviations: LN, lymph node; DSS, disease-specific survival

Spearman correlation analysis among tumor microenvironment markers revealed several significant interrelationships. CD8 + T-cell density showed strong positive correlations with both CD68 + macrophage density (ρ = 0.650, *p* < 0.01) and PDL1 CPS (ρ = 0.602, *p* < 0.01). A moderate positive correlation was also observed between CD68 + density and PDL1 CPS (ρ = 0.400, *p* < 0.05). Older patient age correlated with a higher Ki67 proliferative index (ρ = 0.452, *p* < 0.05) and a lower PDL1 CPS (ρ = -0.436, *p* < 0.05). No other significant correlations were observed among these variables (Supplementary Table 4).

Multivariable logistic regression analysis was performed to identify independent predictors for four distinct clinical outcomes. For local recurrence, several factors retained significant independent associations. Each additional year of age was associated with a markedly lower risk of recurrence by 8.4% (OR: 1.084, 95% CI: 1.017–1.155; *p =* 0.013). A higher Ki67 proliferative index was a strong predictor (OR: 149.95 per 1% increase, 95% CI: 1.014–22168.131; *p =* 0.049), though this estimate had an extremely wide confidence interval. In contrast, each unit increase in CD8 + and CD68 + cell density was associated with a modest but significant reduction in the odds of recurrence (OR: 0.994, *p =* 0.021 and OR: 0.989, *p =* 0.014, respectively). Positive PDL1 expression appeared to be associated with a lower risk of recurrence (OR: 0.061, 95% CI: 0.006–0.600; *p =* 0.017). For the outcomes of lymph node metastasis, distant metastasis, and disease-specific survival, no variables demonstrated statistically significant independent associations in the adjusted models. It is noted that models for several predictors (e.g., HPV coinfection, pleomorphism) did not converge (NC†) or yielded unstable estimates with vast confidence intervals, primarily due to quasi-complete separation or an insufficient number of events (Table 5).

**Table 5:** Multivariable logistic regression analysis of factors associated with clinical outcomes in HMSC

Predictor	Local Recurrence	Lymph Node Metastasis (LNM)	Distant Metastasis (DM)	DSS (Disease-Specific Survival)
Age (per 1 year)	1.084 (1.017–1.155); *p* = 0.013	1.026 (0.969–1.086); *p* = 0.376	1.002 (0.944–1.063); *p* = 0.950	1.057 (0.960–1.164); *p* = 0.260
Ki67 (per 1% increase)	149.950 (1.014–22168.131); *p* = 0.049*	0.350 (0.004–34.634); *p* = 0.654	3.600 (0.015–844.431); *p* = 0.645	56423.532 (< 0.001–3.45e13); *p* = 0.289*
CD8 + Count (per 1 cell)	0.994 (0.989–0.999); *p* = 0.021	1.002 (0.998–1.007); *p* = 0.333	1.001 (0.996–1.006); *p* = 0.708	0.999 (0.993–1.006); *p* = 0.842
CD68 + Count (per 1 cell)	0.989 (0.981–0.998); *p* = 0.014	1.000 (0.992–1.007); *p* = 0.900	0.999 (0.991–1.007); *p* = 0.837	1.003 (0.991–1.014); *p* = 0.648
Tumor Size (per 1 cm)	1.166 (0.797–1.706); *p* = 0.429	1.289 (0.800–2.076); *p* = 0.298	1.729 (0.937–3.191); *p* = 0.080	1.230 (0.633–2.393); *p* = 0.541
CPS (per 1 unit)	0.838 (0.691–1.017); *p* = 0.074	1.064 (0.994–1.138); *p* = 0.073	1.027 (0.958–1.101); *p* = 0.460	1.020 (0.928–1.120); *p* = 0.685
Mitoses (per 1/2 mm²)	1.024 (0.834–1.257); *p* = 0.821	1.052 (0.821–1.348); *p* = 0.689	0.942 (0.697–1.273); *p* = 0.697	1.191 (0.827–1.714); *p* = 0.348
PDL1 Positive (vs. Negative)	0.061 (0.006–0.600); *p* = 0.017	2.167 (0.299–15.705); *p* = 0.444	1.300 (0.155–10.899); *p* = 0.809	1.091 (0.061–19.630); *p* = 0.953
TNM Stage T3/T4 (vs. T1/T2)	1.633 (0.310–8.607); *p* = 0.563	0.857 (0.117–6.264); *p* = 0.879	1.929 (0.173–21.540); *p* = 0.594	0.643 (0.036–11.631); *p* = 0.765
HPV coinfection (vs. single HPV)	NC†	4.222 (0.485–36.767); *p* = 0.192	6.667 (0.665–66.842); *p* = 0.107	4.750 (0.243–92.970); *p* = 0.305
Pleomorphism Present	0.563 (0.031–10.117); *p* = 0.696	NC†	NC†	NC†
Necrosis Present	1.667 (0.257–10.792); *p* = 0.592	0.441 (0.057–3.421); *p* = 0.434	0.278 (0.031–2.497); *p* = 0.253	0.278 (0.015–5.273); *p* = 0.394
PNI Present	1.875 (0.221–15.930); *p* = 0.565	1.583 (0.129–19.422); *p* = 0.719	NC†	NC†
VI Present	0.833 (0.066–10.553); *p* = 0.888	2.500 (0.180–34.669); *p* = 0.495	NC†	NC†
Adjuvant Treatment	0.500 (0.092–2.730); *p* = 0.423	NC‡; *p* = 0.097 (trend in exact test)	NC†	NC†

* Unstable estimate due to quasi-complete separation (extremely wide confidence interval);

NC† Model did not converge due to quasi-complete separation or insufficient events

NC‡ Model did not converge; p-value from Fisher’s exact test as alternative analysis

Cox proportional hazards regression focused on recurrence-free survival as the primary endpoint, given that it was the most frequent event (*n* = 10). In univariate analysis, older age (HR: 1.042 per year, 95% CI: 1.006–1.079; *p* = 0.020), lower CD68 + macrophage density (HR: 0.473 per 100 cells, 95% CI: 0.262–0.853; *p =* 0.013), and negative PDL1 expression (HR: 0.100, 95% CI: 0.013–0.790; *p =* 0.029) were associated with shorter recurrence-free survival. Due to the limited number of events, multivariate analysis was restricted to bivariate models. The most robust model (Model 1: Age + CD68 + density, χ²=11.583, *p* = 0.003) confirmed both increasing age (HR: 1.043, 95% CI: 1.003–1.084; *p* = 0.033) and lower CD68 + density (HR: 0.445 per 100 cells, 95% CI: 0.224–0.886; *p* = 0.021) as independent predictors of recurrence (Supplementary Table 5).

**Fig. 3:**
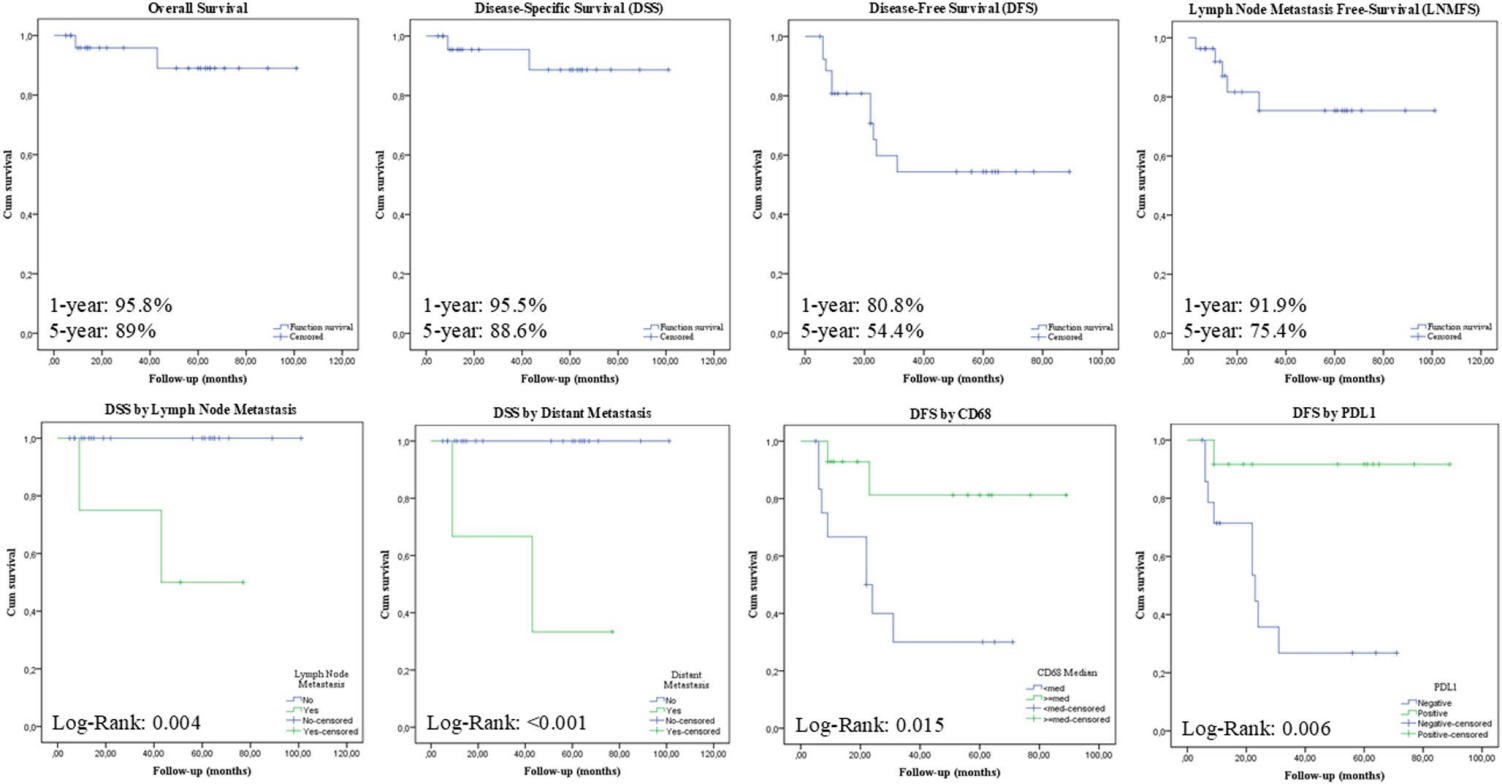
Kaplan Meier analysis

Kaplan-Meier analysis estimated OS rates of 95.8% at 1 year and 89.0% at 5 years. The DSS rate was 95.5% at 1 year and 88.6% at 5 years. For DFS, the rates were 80.8% at 1 year and 54.4% at 5 years. The LNMFS was 91.9% at 1 year and 75.4% at 5 years, while DMFS was 96.3% at 1 year and 78.8% at 5 years. Patients who developed lymph node metastasis had significantly worse DSS than those without (5-year DSS: 50.0% vs. 100.0%; log-rank *p* = 0.004). Distant metastasis was associated with a more pronounced DSS reduction (5-year DSS: 33.3% vs. 100.0%; *p* < 0.001). For DFS, positive PDL1 expression (5-year DFS: 91.7% vs. 26.8%; *p* = 0.006) and a high CD68 + macrophage density (5-year DFS: 81.3% vs. 30.0%; *p* = 0.015) were favorable prognostic factors (Fig. 3).

## Discussion

This study provides a novel contribution by evaluating the impact of PDL1 expression and the inflammatory microenvironment on the prognosis of HMSC patients. Our results suggest that higher densities of inflammatory cells, particularly CD8 + T lymphocytes and CD68 + macrophages, may be associated with a protective effect against recurrence, highlighting the prognostic relevance of tumor immune contexture in HMSC.

The association between inflammatory cell density and recurrence may represent a clinically relevant factor, as HMSC is characterized by frequent recurrence, a low 5-year disease-free survival (DFS) rate of 36.8%, and the need for repeated surgical interventions, which may negatively impact patient quality of life [2, 17]. Moreover, recurrence has been suggested as a potential risk factor for distant metastases in HMSC, yet reliable parameters for risk stratification remain lacking [2].

The distribution of CD8 + T-cell density in our cohort contrasts with that reported for other sinonasal tract malignancies. We observed a nearly balanced distribution between low (48.1%) and high (51.8%) levels of CD8 + T-cell infiltration, differing from the more variable rates of high CD8 + infiltrate in sinonasal squamous cell carcinoma (SCC) (9.2–56.6%) and exceeding the proportions reported for sinonasal mucosal melanoma (29.6%) and intestinal-type adenocarcinoma (ITAC) (8%) [7, 9, 18–20]. Although high CD8 + density is generally associated with favorable prognoses in sinonasal tumors, its impact varies by histological subtype. In SCC, elevated CD8 + infiltration correlates with improved overall survival (OS) and DFS, though its association with lymph node metastasis was reported [7, 20]. In ITAC, high CD8 + levels are consistently linked to reduced recurrence, lower metastatic rates, and enhanced OS [18]. In sinonasal mucosal melanoma, high CD8 + infiltration improves OS without significantly affecting recurrence rates [19].

Our findings are consistent with the well-established relationship between immune infiltration and PDL1 expression in sinonasal carcinomas. We observed a strong positive correlation between CD8 + T-cell density and PDL1 expression, with CD8 + count emerging as a significant predictor of PDL1 status [7, 9, 20]. Tumors with robust infiltration of CD8 + T cells and CD68 + macrophages tended to exhibit improved local control, indicating a coordinated, albeit checkpoint-inhibited, anti-tumor response effective in restraining disease progression [7, 18, 20].

CD8 + T cells act as central mediators of antitumor immunity, directing lysing tumor cells via perforin–granzyme release and Fas–FasL interactions, while also secreting cytokines such as IFN-γ and TNF-α to amplify immune responses [21]. Macrophages display high plasticity and can adopt divergent functional phenotypes, with M1 macrophages promoting antitumor immunity through phagocytosis, antigen presentation, and pro-inflammatory cytokines production, whereas M2 macrophages drive immunosuppression, angiogenesis, and stromal remodeling [22]. Although CD68 is a pan-macrophage marker and does not distinguish between M1 and M2 subsets, the association between higher macrophage density, increased CD8 + T-cell infiltration, and improved clinical outcomes suggests a predominance of M1-like polarized macrophages in HMSC. Nevertheless, future studies using polarization-specific markers are needed to confirm this hypothesis. This observation raises the possibility that PDL1-positive tumors might represent potential candidates for immunotherapy.

Our analysis revealed nuanced relationships within the HMSC microenvironment. PDL1 positivity was associated with a robust CD8 + T-cell infiltrate and, consistent with this inflamed microenvironment, with reduced recurrence risk. This apparent paradox may be explained by PDL1 expression serving as a biomarker of pre-existing anti-tumor immune responses, specifically an “inflamed” tumor microenvironment, rather than solely as a mechanism of immune escape [23, 24]. In this context, PDL1 upregulation likely represents adaptive immune resistance, where tumors attempt to counteract an otherwise effective immune attack. Conversely, tumors lacking PDL1 expression tended to exhibit higher proliferative indices and lower immune infiltration, suggesting a potential “immune-cold” phenotype that may be driven more by cell-intrinsic proliferation than adaptive immune evasion [23, 25]. This observed dichotomy aligns with emerging concepts in cancer immunology wherein PDL1 expression can reflect either immune activation or suppression, contingent upon the broader immune context.

Age appeared to influence tumor behavior, as younger patients displayed higher PDL1 expression, whereas older patients presented higher proliferative indices and increased recurrence, reflecting complex age-related interactions between proliferation, immune infiltration, and checkpoint regulation that should be considered in treatment planning. Overall, these observations suggest that PDL1 expression alone may not to be a straightforward prognostic marker and highlight the necessity of evaluating it within the broader tumor immune context to identify patients most likely to benefit from immunotherapy and those at risk for recurrence. Consistently, studies in non-small cell lung cancer and esophageal SCC have linked high Ki67 levels to reduced clinical response to anti-PD-1 therapy and poorer survival, suggesting a connection between proliferation and T-cell exhaustion [26].

Our study presented some limitations. The relatively small sample size (*n* = 27) restricts statistical power for complex multivariable analyses and may have contributed to the convergence issues observed in logistic regression models. Nonetheless, the observed effect sizes for significant associations were generally large, indicating clinical relevance despite sample size constraints. The non-normal distribution of key variables (Ki67, CD8 + count, follow-up time) necessitated non-parametric approaches, which may have reduced statistical power compared to parametric alternatives. However, the robust non-parametric methods employed provide valid statistical inference regardless of distributional assumptions. Additionally, the multiple comparisons inherent in comprehensive correlation and association testing increase the risk of type I error. However, the combined consideration of effect sizes, statistical significance, and biological plausibility supports the validity of our principal findings. Overall, these results provide valuable insights into the immune microenvironment of HMSC and highlight important associations between immune markers, tumor characteristics, and clinical outcomes that warrant validation in larger prospective studies.

## Conclusion

Our findings underscore the complex and inflammation-rich microenvironment of HMSC. Elevated densities of CD8 + T-cells and CD68 + macrophages were associated with improved clinical outcomes, including reduced recurrence rates. A strong positive correlation was observed between CD8 + T-cell and CD68 + macrophage infiltration. Furthermore, PDL1 expression was positively correlated with CD8 + T-cell density and was more frequently detected in younger patients. An inverse trend was noted between PDL1 expression and the Ki67 proliferation index. Collectively, these results highlight the prognostic relevance of the immune microenvironment and patient age in HMSC, suggesting that anti-PDL1 immunotherapy may be considered for selected cases, particularly those exhibiting an inflamed tumor phenotype.

## Supplementary Information

Below is the link to the electronic supplementary material.

Supplementary Material 1

Supplementary Material 2

Supplementary Material 3

Supplementary Material 4

Supplementary Material 5

## Data Availability

No datasets were generated or analysed during the current study.
