# Supplementary material for: Characterization of CD8 + and CD68 + Microenvironment and PDL1 Expression in HPV-related Multiphenotypic Sinonasal Carcinoma
Source: Head Neck Pathol. 2026 Mar 19;20(1):34. doi: 10.1007/s12105-026-01908-0 (PMC13003031; doi:10.1007/s12105-026-01908-0)
Supplement: Supplementary file 1 — Supplementary Material 1 [file 12105_2026_1908_MOESM1_ESM.docx]

**Supplementary Table 2.** Histopathological features of 27 HMSC patients included in this study.

| **Case** | **Epithelial dysplasia** | **Mitoses per 2mm2** | **Pleomorphism** | **Necrosis** | **PNI** | **VI** | **Cell appearance** | **Nuclear appearance** | **Arrangement** | **Stroma** |
| --- | --- | --- | --- | --- | --- | --- | --- | --- | --- | --- |
| 1 | 6 - 8 layers | 7 | Yes | No | No | No | Basophilic, clear | Round to oval hyperchromatic and indistinct nucleoli | Tubular, Cords | Myxohyaline |
| 2 | 3 - 4 layers | 11 | Yes | Yes | No | No | Clear, Basophilic, spindle | Round to oval with dispersed chromatin | Tubular, Cords | Myxoid |
| 3 | 2 - 3 layers | 3 | Yes | Yes | No | No | Basophilic, spindle, clear | Round to oval hyperchromatic and indistinct nucleoli | Solid, Nests | Myxoid |
| 4 | 10 - 12 layers | 7 | Yes | Yes | No | No | Clear, Basophilic, spindle | Round to oval hyperchromatic and a small distinct nucleoli | Tubular, Cords | Myxoid |
| 5 | 2 - 3 layers | 11 | Yes | No | No | No | Eosinophilic, Basophilic, clear | Round to oval with dispersed chromatin | Cribriform | Myxohyaline |
| 6 | 2 - 3 layers | 3 | Yes | Yes | No | No | Clear, spindle, eosinophilic | Round to oval with dispersed chromatin | Solid, Cribriform | Myxohyaline |
| 7 | 2 - 3 layers | 15 | No | Yes | No | No | Basophilic, eosinophilic, spindle | Round to oval with dispersed chromatin | Solid, Single cells | Hyaline |
| 8 | 2 - 3 layers | 6 | Yes | Yes | No | No | Basophilic, clear, spindle, and eosinophilic | Round to oval hyperchromatic and a small distinct nucleoli | Cribriform | Hyaline |
| 9 | 3 - 4 layers | 5 | Yes | Yes | No | No | Basophilic, clear | Round to oval hyperchromatic and a small distinct nucleoli | Solid, Cribriform | Myxoid |
| 10 | 10 - 12 layers | 17 | No | Yes | Yes | Yes | Basophilic, spindle, clear | Round to oval with dispersed chromatin | Solid, Ribbon-like | Hyaline |
| 11 | 10 - 12 layers | 9 | Yes | No | No | No | Basophilic, clear, spindle, and eosinophilic | Round to oval with dispersed chromatin | Solid | Myxohyaline |
| 12 | 6 - 8 layers | 14 | Yes | Yes | Yes | Yes | Basophilic, spindle, clear | Round to oval with dispersed chromatin | Cribriform | Myxohyaline |
| 13 | 6 - 8 layers | 11 | Yes | Yes | No | No | Basophilic, clear | Round to oval hyperchromatic and a small distinct nucleoli | Solid, Ribbon-like | Hyaline |
| 14 | 10 - 12 layers | 5 | Yes | Yes | No | Yes | Basophilic, spindle, clear | Round to oval with dispersed chromatin | Tubular, Cords | Myxoid |
| 15 | 2 - 3 layers | 6 | Yes | Yes | No | No | Basophilic, clear, spindle, and eosinophilic | Round to oval hyperchromatic and a small distinct nucleoli | Cribriform, glomeruloid-like | Hyaline |
| 16 | 3 - 4 layers | 5 | Yes | Yes | No | No | Basophilic, eosinophilic, spindle | Round to oval with dispersed chromatin | Cribriform | Myxoid |
| 17 | 6 - 8 layers | 15 | Yes | Yes | Yes | No | Basophilic | Round to oval hyperchromatic and a small distinct nucleoli | Solid, Cribriform | Hyaline |
| 18 | 8 - 10 layers | 8 | Yes | Yes | No | No | Basophilic, spindle, clear | Round to oval hyperchromatic and indistinct nucleoli | Tubular, Cords | Myxoid |
| 19 | 8 - 10 layers | 4 | Yes | Yes | No | No | Eosinophilic | Round to oval hyperchromatic and a small distinct nucleoli | Tubular, Cords | Hyaline |
| 20 | 6 - 8 layers | 7 | Yes | No | No | No | Basophilic, spindle, clear | Round to oval hyperchromatic and indistinct nucleoli | Solid, Single cells | Hyaline |
| 21 | 15 - 20 layers | 5 | Yes | No | No | No | Basophilic, spindle | Round to oval hyperchromatic and indistinct nucleoli | Solid, Single cells | Myxohyaline |
| 22 | 5 - 6 layers | 7 | Yes | No | No | No | Basophilic | Round to oval hyperchromatic and a small distinct nucleoli | Tubular, Cords | Myxoid |
| 23 | 2 - 3 layers | 5 | Yes | Yes | No | No | Clear, spindle, eosinophilic | Round to oval with dispersed chromatin | Tubular, Cords | Hyaline |
| 24 | 2 - 3 layers | 6 | Yes | Yes | No | No | Basophilic, spindle | Round to oval hyperchromatic and indistinct nucleoli | Cribriform | Myxoid |
| 25 | 12 - 15 layers | 11 | Yes | Yes | No | No | Basophilic, eosinophilic, spindle | Round to oval hyperchromatic and indistinct nucleoli | Tubular, Cords | Myxoid |
| 26 | 12 - 15 layers | 11 | Yes | No | No | No | Basophilic, eosinophilic, spindle | Round to oval hyperchromatic and indistinct nucleoli | Cribriform | Hyaline |
| 27 | 5 - 6 layers | 7 | Yes | Yes | Yes | No | Basophilic, clear | Round to oval with dispersed chromatin | Solid, Single cells | Hyaline |
| PNI: perineural invasion; VI: vascular invasion | | | | | | | | | | |
