# Supplementary material for: Characterization of CD8 + and CD68 + Microenvironment and PDL1 Expression in HPV-related Multiphenotypic Sinonasal Carcinoma
Source: Head Neck Pathol. 2026 Mar 19;20(1):34. doi: 10.1007/s12105-026-01908-0 (PMC13003031; doi:10.1007/s12105-026-01908-0)
Supplement: Supplementary file 2 — Supplementary Material 2 [file 12105_2026_1908_MOESM2_ESM.docx]

**Supplementary Table 1.** Clinicodemographic features of 27 HMSC patients included in this study.

| **Case** | **Age** | **Sex** | **Location** | **Tumor size (cm)** | **Treatment** | **TNM (stage)** | **Recurrence** | **Lymph Node Meta** | **Distant Meta** | **HPV genotype** | **Followup** | **Time (mo)** | |
| --- | --- | --- | --- | --- | --- | --- | --- | --- | --- | --- | --- | --- | --- |
| 1 | 36 | F | Paranasal sinus | 2.03 | Surgery | pT2 N0 M0 (II) | No | No | No | 33 | AWoD | 60 | |
| 2 | 81 | F | Paranasal sinus | 5.76 | Surgery | pT3 N0 M0 (III) | Yes (23 mo) | No | No | 33 | AWoD | 101 | |
| 3 | 45 | M | Nasal cavity | 3.34 | Surgery + Radiotherapy | pT3 N0 M0 (III) | No | No | No | 35 | AWoD | 71 | |
| 4 | 75 | M | Paranasal sinus | 4.13 | Surgery | pT2 N0 M0 (II) | Yes (9 mo) | No | No | 33 | AWoD | 15 | |
| 5 | 69 | F | Nasal cavity | 1.3 | Surgery + Radiotherapy | pT1 N0 M0 (I) | Yes (22 mo) | No | No | 33 | AWoD | 67 | |
| 6 | 28 | F | Nasal cavity | 7.98 | Surgery + Chemoradiotherapy | pT4a N0 M0 (IVA) | No | Yes (11 mo) | Yes (48 mo, lung) | 33, 51 | AWoD | 77 | |
| 7 | 57 | F | Nasal cavity | 2.28 | Surgery | pT2 N0 M0 (II) | No | No | No | 16 | AWoD | 63 | |
| 8 | 33 | M | Paranasal sinus | 3.33 | Surgery + Chemoradiotherapy | pT2 N0 M0 (II) | No | No | No | 33 | AWoD | 56 | |
| 9 | 38 | F | Nasal cavity | 5.22 | Surgery + Radiotherapy | pT3 N0 M0 (III) | No | No | No | 26, 33 | AWoD | 65 | |
| 10 | 79 | F | Paranasal sinus | 5.04 | Surgery | pT3 N0 M0 (III) | Yes (31 mo) | No | No | 33 | AWoD | 65 | |
| 11 | 56 | F | Nasal cavity | 8.12 | Surgery + Radiotherapy | pT4a N0 M0 (IVA) | No | No | No | 33 | AWoD | 64 | |
| 12 | 90 | F | Paranasal sinus | 1.3 | Surgery + Radiotherapy | pT1 N0 M0 (I) | No | Yes (29 mo) | No | 33 | AWoD | 51 | |
| 13 | 88 | F | Paranasal sinus | 8.26 | Surgery + Radiotherapy | pT4b N0 M0 (IVB) | Yes (24 mo) | Yes (16 mo) | Yes (24 mo, lung) | 52 | DOD | 43 | |
| 14 | 45 | M | Paranasal sinus | 3.35 | Surgery + Radiotherapy | pT3 N0 M0 (III) | No | No | No | 16, 33 | AWoD | 89 | |
| 15 | 37 | M | Nasal cavity | 3.9 | Surgery | pT3 N0 M0 (III) | No | No | No | 33 | AWoD | 61 | |
| 16 | 77 | F | Paranasal sinus | 1.3 | Surgery + Radiotherapy | pT3 N0 M0 (III) | Yes (9 mo) | No | No | 33 | AWoD | 13 | |
| 17 | 88 | M | Paranasal sinus | 3.15 | Surgery | pT2 N0 M0 (II) | No | No | No | 35 | AWoD | 10 | |
| 18 | 51 | M | Paranasal sinus | 6.3 | Surgery + Radiotherapy | pT3 N0 M0 (III) | No | No | No | 33 | AWoD | 19 | |
| 19 | 68 | M | Nasal cavity | 5.65 | Surgery | pT3 N0 M0 (III) | Yes | No | No | 33 | AWD | 29 | |
| 20 | 50 | F | Nasal cavity | 3.9 | Radiotherapy | pT3 N0 M0 (III) | No | No | No | 33 | AWoD | 14 | |
| 21 | 63 | F | Paranasal sinus | 6.44 | Surgery + Chemoradiotherapy | pT3 N0 M0 (III) | Yes (7 mo) | Yes (14 mo) | Yes (14 mo, bone) | 33 | AWD | 14 | |
| 22 | 41 | M | Nasal cavity | 6.02 | Surgery + Radiotherapy | pT3 N0 M0 (III) | No | No | No | 18, 52 | AWoD | 22 | |
| 23 | 77 | M | Nasal cavity | 4.62 | Surgery + Radiotherapy | pT3 N0 M0 (III) | No | No | No | 33 | AWoD | 11 | |
| 24 | 60 | M | Nasal cavity | 3.92 | Surgery + Radiotherapy | pT2 N0 M0 (II) | Yes (6 mo) | No | No | 35 | AWoD | 7 | |
| 25 | 51 | F | Paranasal sinus | 1.3 | Surgery + Radiotherapy | pT1 N0 M0 (I) | No | No | No | 33 | AWoD | 5 | |
| 26 | 61 | F | Nasal cavity | 1.69 | Surgery + Chemoradiotherapy | pT1 N0 M0 (I) | No | Yes (3 mo) | Yes (3 mo, liver) | 33, 51 | DOD | 9 | |
| 27 | 62 | M | Paranasal sinus | 4.73 | Surgery + Radiotherapy | pT3 N0 M0 (III) | Yes (6 mo) | No | No | 33 | AWoD | 7 | |
| F: female; M: male; mo: months; AWoD: alive without disease; AWD: alive with disease; DOD: dead of disease; Meta: metastasis, cm: centimeter. | | | | | | | | | | | | |  |
