# Supplementary material for: Characterization of CD8 + and CD68 + Microenvironment and PDL1 Expression in HPV-related Multiphenotypic Sinonasal Carcinoma
Source: Head Neck Pathol. 2026 Mar 19;20(1):34. doi: 10.1007/s12105-026-01908-0 (PMC13003031; doi:10.1007/s12105-026-01908-0)
Supplement: Supplementary file 3 — Supplementary Material 3 [file 12105_2026_1908_MOESM3_ESM.docx]

**Supplementary Table 3.** Inflammatory microenvironment features of HMSC patients included in this study.

| **Case** | **Ki67 (fraction** | **Ki67 split (1=<med; 2=>=med)** | **PDL1 (1=neg; 2=pos)** | **PDL1 CPS** | **CD8** | **CD8 median (1=<med; 2=>=med)** | **CD68** | **CD68 median (1=<med; 2=>=med)** |
| --- | --- | --- | --- | --- | --- | --- | --- | --- |
| 1 | 0.1966 | 1 | 2 | 35.9 | 876 | 2 | 456 | 2 |
| 2 | 0.6915 | 2 | 1 | 0 | 450 | 1 | 402 | 2 |
| 3 | 0.4592 | 1 | 1 | 0 | 381 | 1 | 344 | 1 |
| 4 | 0.3507 | 1 | 2 | 11.8 | 409 | 1 | 228 | 1 |
| 5 | 0.5655 | 2 | 1 | 0.4 | 335 | 1 | 190 | 1 |
| 6 | 0.0575 | 1 | 2 | 30.4 | 946 | 2 | 435 | 2 |
| 7 | 0.346 | 1 | 2 | 11.3 | 752 | 2 | 410 | 2 |
| 8 | 0.5678 | 2 | 1 | 0 | 229 | 1 | 566 | 2 |
| 9 | 0.2445 | 1 | 2 | 8.3 | 453 | 2 | 172 | 1 |
| 10 | 0.7298 | 2 | 1 | 0.4 | 339 | 1 | 231 | 1 |
| 11 | 0.6273 | 2 | 1 | 0 | 403 | 1 | 554 | 2 |
| 12 | 0.1149 | 1 | 2 | 45.9 | 864 | 2 | 387 | 2 |
| 13 | 0.6692 | 2 | 1 | 0 | 227 | 1 | 283 | 1 |
| 14 | 0.6906 | 2 | 2 | 27.5 | 743 | 2 | 398 | 2 |
| 15 | 0.3159 | 1 | 2 | 14.7 | 640 | 2 | 325 | 1 |
| 16 | 0.7485 | 2 | 1 | 0 | 451 | 1 | 522 | 2 |
| 17 | 0.6963 | 2 | 1 | 0.7 | 722 | 2 | 564 | 2 |
| 18 | 0.6668 | 2 | 2 | 19.1 | 789 | 2 | 548 | 2 |
| 19 | 0.447 | 1 | 1 | 0 | 665 | 2 | 316 | 1 |
| 20 | 0.1415 | 1 | 2 | 5.9 | 417 | 1 | 482 | 2 |
| 21 | 0.6911 | 2 | 1 | 0 | 409 | 1 | 149 | 1 |
| 22 | 0.3586 | 1 | 2 | 33.8 | 925 | 2 | 245 | 1 |
| 23 | 0.5998 | 2 | 1 | 0.7 | 321 | 1 | 455 | 2 |
| 24 | 0.4381 | 1 | 1 | 0.8 | 463 | 2 | 304 | 1 |
| 25 | 0.2982 | 1 | 1 | 0.4 | 881 | 2 | 342 | 1 |
| 26 | 0.6811 | 2 | 2 | 30.1 | 855 | 2 | 562 | 2 |
| 27 | 0.5741 | 2 | 1 | 0 | 441 | 1 | 122 | 1 |
|  |  |  |  |  |  |  |  |  |
