# Supplementary material for: Characterization of CD8 + and CD68 + Microenvironment and PDL1 Expression in HPV-related Multiphenotypic Sinonasal Carcinoma
Source: Head Neck Pathol. 2026 Mar 19;20(1):34. doi: 10.1007/s12105-026-01908-0 (PMC13003031; doi:10.1007/s12105-026-01908-0)
Supplement: Supplementary file 4 — Supplementary Material 4 [file 12105_2026_1908_MOESM4_ESM.docx]

**Supplementary Table 4.** Interrelationships among age, proliferation, and tumor immune microenvironment markers in HMSC.

| **Ki67** | **CD8+** | **CD68+** | **CPS (PDL1)** |  |
| --- | --- | --- | --- | --- |
| Age | 0.452* | -0.150 | -0.250 | -0.436* |
| Ki67 | — | 0.300 | -0.100 | -0.350 |
| CD8+ | 0.300 | — | 0.650** | 0.602** |
| CD68+ | -0.100 | 0.650** | — | 0.400* |
| CPS (PD-L1) | -0.350 | 0.602** | 0.400* | — |
| Spearman's correlation coefficients (ρ). *p<0.05, **p<0.01. CD8+ = cytotoxic T lymphocytes; CD68+ = macrophages; CPS = Combined Positive Score for PD-L1. | | | | |
