# Supplementary material for: Characterization of CD8 + and CD68 + Microenvironment and PDL1 Expression in HPV-related Multiphenotypic Sinonasal Carcinoma
Source: Head Neck Pathol. 2026 Mar 19;20(1):34. doi: 10.1007/s12105-026-01908-0 (PMC13003031; doi:10.1007/s12105-026-01908-0)
Supplement: Supplementary file 5 — Supplementary Material 5 [file 12105_2026_1908_MOESM5_ESM.docx]

**Supplementary Table 5**. Univariate and bivariate Cox regression analyses of factors associated with DFS.

| **Variable** | **Univariate Analysis HR (95% CI)** | **p-value** | **Multivariate Models** |
| --- | --- | --- | --- |
| Age (per year) | 1.042 (1.006-1.079) | **0.020** | Model 1: 1.043 (1.003-1.084); *p =* 0.033 Model 2: 1.038 (1.000-1.077); p = 0.049 Model 3: 1.032 (0.992-1.074); p = 0.120 |
| CD68+ density (per 100 cells) | 0.473 (0.262-0.853) | **0.013** | Model 1: 0.445 (0.224-0.886); p = 0.021 Model 5: 0.472 (0.259-0.859); p = 0.014 |
| CD8+ density (per 100 cells) | 0.721 (0.516-1.006) | 0.054 | Model 2: 0.773 (0.557-1.072); p = 0.123 Model 4: 1.003 (0.625-1.609); p = 0.990 |
| PD-L1 expression (positive vs negative) | 0.100 (0.013-0.790) | **0.029** | Model 3: 0.152 (0.018-1.261); p = 0.081 Model 4: 0.099 (0.007-1.468); p = 0.093 |
| Ki67 index (per 1% increase) | 38.191 (0.808-1805.729) | 0.064 | Model 5: 46.048 (1.060-2001.215); p = 0.047 |
| Tumor size (per cm) | 1.055 (0.797-1.396) | 0.710 | - |
| CPS score (per point) | 0.870 (0.744-1.018) | 0.083 | - |
| Model 1: Age + CD68+ density (χ²=11.583, p = 0.003) Model 2: Age + CD8+ density (χ²=8.442, p = 0.015) Model 3: Age + PD-L1 expression (χ²=9.614, p = 0.008) Model 4: CD8+ density + PD-L1 expression (χ²=7.229, p = 0.027) Model 5: Ki67 index + CD68+ density (χ²=12.041, p = 0.002) Abbreviations: CI, confidence interval; CPS, combined positive score; HR, hazard ratio. Interpretation: Bolded HRs and p-values indicate statistical significance (p<0.05). Multivariate models were limited to 2 predictors each due to the small number of events (n=10 recurrences). The most robust model (Model 1) identified age and CD68+ macrophage density as independent predictors of recurrence. | | | |
